# Does reality meet expectations? An analysis of medical students’ expectations and perceived learning during mandatory research projects

**DOI:** 10.1186/s12909-019-1526-x

**Published:** 2019-03-29

**Authors:** Riitta Möller, Maria Shoshan

**Affiliations:** 1 Department of Medical Epidemiology and Biostatistics, Karolinska Institutet, Nobelsväg 12 a, 171 77 Stockholm, Sweden; 2 Departments of Oncology-Pathology and Medical Epidemiology and Biostatistics, Stockholm, Sweden

**Keywords:** Medical students, Scholarly projects, Undergraduate research, Student thesis, Research activities

## Abstract

**Background:**

Although much has been written about structure and outcomes of medical students’ curricular research projects, less attention has been paid to the expectations on such projects. In order to foster students’ scientific understanding and improve the quality of mandatory research projects, we compared students’ pre-course expectations with their post-course insights regarding learning and transferable skills.

**Methods:**

A prospective cross-sectional questionnaire study. All students registered on a mandatory 20-week research project course in 2011–2013 were e-mailed questionnaires in the beginning and after the course asking them to rate statements on expectations and perceived learning on a 5-point Likert scale. Of 652 students, 358 (mean age 26 years; range 21–49; 63% females) returned both questionnaires, corresponding to a response rate of 55%.

**Results:**

The ratings for expectations as well as perceived learning were highest for learning to search and critically appraise literature. The greatest pre- and post-course differences were indicated for participation in scientific discussions and oral communication. Surprisingly, both pre- and post-course ratings were low for research ethics. The highest post-course ratings regarding skills for future working life were given to items pertaining to understanding the scientific basis of medicine, ability to follow the development of knowledge and to critically integrate knowledge. Female students had higher expectations than male students. Those with a previous university degree had lower ratings of expectations and perceived learning. Students with basic science projects reported higher expectations and higher learning compared to students with other projects. Previous research experience had no significant influence on expectations nor learning. The correlations between post-course ratings of learning and skills showed that problem-solving ability had a relatively high correlation with all skills.

**Conclusions:**

Students had high expectations and perceived the course improved crucial practical skills. However, expectations were not quite met regarding aspects of scientific communication, and hypothesis formulation, likely because these require more extensive practice and feedback. Students should be actively involved in ethical discussions and oral communication should be trained repeatedly as it is an important task of doctors to communicate scientific information to patients and non-experts.

**Electronic supplementary material:**

The online version of this article (10.1186/s12909-019-1526-x) contains supplementary material, which is available to authorized users.

## Background

Evidence-based medicine (EBM) aims to optimize clinical decision-making based on evidence from well-conducted research. Applying EBM entails identification of relevant problems and questions, searching and evaluating current scientific literature, and applying the findings into practice [1]. Thus, in order to provide high-quality health care, the skills of searching, reading and critically appraising literature have to be developed already during undergraduate education [2]. Consequently, an increasing number of medical schools worldwide have integrated formal training in scientific and research related skills within the core curricula [3].

Despite trends in undergraduate education towards greater integration of subjects, the curricula are often organized in specialized fields such as anatomy and physiology. This also applies to research skills. Prior to undertaking research projects, students are usually offered separate or partly integrated courses in e.g., epidemiology and research methodology. These courses oftentimes focus on “using research”, e.g., training practical skills such as carrying out a literature search or appraising evidence rather than “research training” (e.g. formulating a research question, and critically analyzing and drawing conclusions from research data) [4, 5]. Thus, medical students who embark on scholarly projects often lack the experience to carry out an entire research process and may find it difficult to understand the generic value of the course in regard to their future career and life-long learning [6].

The impact of research training is not fully understood. Over the past decades many publications have discussed the structure of students’ scholarly projects, their administration and outcomes [7–9]. Only a few studies have used a pre/post research design to explore students’ learning during research training. DeHaven and Chen [10] evaluated a 9-week elective summer research program in which 9 students participated. The results showed that students learned most about ethical reviews and research processes. In another study, 11 first-year medical students who participated in a summer research assistantship completed a questionnaire and an interview [11]. This study found that students’ confidence in designing and performing clinical studies increased. In addition, some students reported enhanced technical skills. Mullan et al. [12] carried out a self-assessment study among 200 medical students who completed a questionnaire before and after an individual research project embedded in a 12-month clinical placement. Their study showed increases in scores from pre- to post-placement in writing a research proposal, writing and presenting a research report as well as analyzing and interpreting results. However, there are no studies that explore students’ expectations on individual research projects, especially in relation to their own post-course perceptions of what skills they have actually gained. Identification of what students expect and then experience can help educators towards a better alignment of teaching and learning activities and supervision, thus promoting the quality of education [13]. This is particularly important when the research projects are mandatory and students’ interest in research may vary.

The aim of this study was to examine the medical students’ pre-course expectations and post-course perceptions of acquired knowledge and skills. Specifically, we wanted to shed light on (i) what are medical students’ preconceptions of research skills and tools, and (ii) which types of knowledge and skills the students themselves felt the course had had an impact on. Understanding students’ expectations is a key starting point for optimizing transformed teaching and learning practices. This is particularly important for structuring mandatory research projects since the students often have quite varying levels of interest and experience in research.

## Methods

### Design and setting of the study

This is a questionnaire-based cross-sectional study. The context is an undergraduate medical program (Karolinska Institutet, KI), which consists of 11 semesters, each comprising 20 weeks, altogether corresponding to 5.5 academic years. The first two years comprise basic sciences (e.g. cell biology and histology) and the last 3.5 years mainly clinical education (e.g. medicine, surgery and pediatrics). The curriculum includes a so-called Scientific Development thread, or theme, that runs throughout semesters 1–9. Before taking the research project course in semester 7, the participants in the present study received lectures and seminars on information searching, philosophy of science, medical ethics, EBM, study design and statistics. These activities also included 3 mandatory assignments: 1) a short oral and a written presentation of a hypothesis based on 3 MeSH terms they were assigned individually (semester 1); 2) a written assignment including interpretation of statistical analysis from a scientific paper (semester 4); and 3) a written assignment comprising a presentation of a clinical problem, development of a research question using the PICO (patient, intervention, comparison, outcome) model, literature search and analysis of the results (semester 5). The total time for the teaching and learning activities pertaining to Scientific Development during semesters 1–6, i.e., before students take the research project course, corresponds to 2 weeks.

It is expected that after the mandatory research course (7th semester; 20 weeks) students should have acquired a deeper understanding of the scientific basis of medicine and be able to interpret and evaluate scientific literature. To this end, the students individually plan and carry out a research project and present a research report essentially formatted as a scientific publication. The course includes only little face-to-face teaching by the faculty; instruction is instead given by supervisor/−s and their research teams. Supervisors are active researchers with at least a PhD degree who can offer a suitable project in their area of expertise. However, the progress of each project is monitored by a research-active faculty coordinator with at least a PhD degree. Each coordinator is responsible for approximately 10–15 students per semester and arranges three seminars (project plan, half-time, and examination) during which each student has to give an oral presentation of her/his own project and get feedback based on criteria. Thus, each coordinator acts as a tutor and examining teacher during the seminars. The final version of the report is not assessed by each student’s own coordinator but by examiners who are senior researchers and know the learning outcomes as well as the structure of the course. The total number of examiners is approximately 6–8 and care is taken to ensure that their evaluations are based on consensual criteria. On average 85% of the students pass the examination on the first occasion. The two authors of this paper (RM, MS) were course directors and coordinator (MS) for the course during the time of the study.

### Participants

In total 651 medical students who attended the research project course between 2011 through 2013 were eligible to participate. An explanatory statement outlining the aims, method and voluntary nature of project was provided to students both verbally and in written format at the course start. The two questionnaires – one at course start and the other at the end of the course - were distributed by e-mail. In case of no reply, two reminders were sent 2–3 weeks after the first submission.

### Data collection methods

Data were retrieved with two on-line questionnaires developed specifically for this survey and partly based on a previous study on PhD students’ research experiences [14] as there are no cross-culturally validated instruments to evaluate students’ learning in research environments in the Swedish context. There were 4 main sections in the questionnaire: 1) sociodemographic data; 2) previous studies; 3) expectations on the course (pre-course questionnaire) and 4) perceptions of learning (post-course questionnaire). Sociodemographic background data included gender, age (categorized as <=22, 23–26, > = 27 years) and educational background (previous university degree or not). Previous university degree was considered important because all higher education degrees in Sweden comprise a mandatory degree (research) project. Previous research experience was defined as previous work (of at least 1 month) in a research group or attending an extracurricular research preparatory course (semester 1–4) for medical students at our university. Responders were asked to rate statements regarding their expectations and perceptions of learning on a 5-point Likert scale, where each of the statements were on a scale of 1 (low priority; not at all) to 5 (high priority; to a very large extent). In Additional file 1: Questionnaires, the statements listed, e.g., skills related to research activities (in pre- and post-course questionnaires) and future working life (in the post-course questionnaire). The questionnaire was compiled by the first author and one of the coordinators, and a pilot version was field-tested on the students who were enrolled on the research project course (*n* = 51) in the fall semester of 2010. The final version was created after slight modifications of the wording of some items of the pilot version and based on the comments from students and the specific questionnaire group at our department comprising 2 epidemiologists and a research nurse. No one found the questions upsetting, disturbing, or hard to understand**.** The data regarding type of project were retrieved from students’ final research reports**.**

The differences between students’ expectations and perceptions were computed by subtracting the expectation mean score from the perception mean score. A negative difference indicates that perception scores were lower than expectation scores. Students with the lowest ratings were defined as those who had rated at least two items as 1 on the Likert scale (*n* = 26), and those with highest ratings as those who had rated at least two items as 5 (*n* = 235), respectively. Completion of the questionnaires took approximately 20 min. Each participant received 2 cinema tickets as compensation.

### Statistics

Descriptive statistics were used to characterize data and describe the population features. The Mann-Whitney U test or Welch two sample t-test was used to compare two independent groups while Kruskal-Wallis test was used when more than two independent groups were compared. Non-parametric Wilcoxon signed-rank test was used to compare the groups before and after the course. For post-hoc analyses, Nemenyi tests were performed*.* The level of significance was set to 0.05. Bonferroni correction was used for multiple analyses. To explore the relation between ratings of expectations and experienced learning and age, gender, previous university degree and type of study, we used a multivariable ordinal regression model. The correlation between post-course ratings of learning and skills was calculated using Kendall’s Tau-b. The statistical analyses were performed with R version 3.4.1.

## Results

### Student characteristics

In total, 358 students returned both questionnaires corresponding to a response rate of 55%. Thirteen students answered only one of the questionnaires and were therefore excluded. The demographic characteristics of the study population and the nonresponders are summarized in Table 1. The groups differed from each other regarding gender and age; the non-responders were older and the majority of them were males (*p* < 0.001). In the study population, there were no statistically significant differences between male and female students regarding time spent in research activities before the course (*p* = 0.29), previous university studies (*p* = 0.70), or previous university degree (*p* = 0.93). Those with a previous university degree were on average 9 years older than those without a degree. Students’ research projects were classified as clinical (61%), basic science (22%), epidemiological (10%) or other (7%), e.g., leadership, management, or medical education projects. Female students chose clinical studies more often than did male students (*p* = 0.004).

**Table 1:** Demographic characteristics of the study population and nonresponders

Variables		Study population (*N* = 358)	Non-responders (*N* = 280)
Gender	Female	227 (63%)	127 (45%)*
	Male	131 (37%)	153 (55%)
Age (years)	Mean	26	27*
	Range	21-49	21-42
	> = 22	79 (22%)	21 (7%)
	23–26	188 (53%)	128 (46%)
	> = 27	91 (25%)	131 (47%)
Previous research activities		82 (23%)	Data missing
Previous university studies		186 (52%)	Data missing
Previous university degree		47 (13%)	Data missing
Type of project	Clinical	220 (61%)	141 (50%)
	Basic science	80 (22%)	81 (30%)
	Epidemiology	35 (10%)	29 (10%)
	Other	23 (6%)	29 (10%)

^*^P-value < 0.001

### Expectations on the course

Overall, the students’ expectations on the course were high regarding skills needed to carry out a research project, rather than on acquiring medical knowledge. Thus, the highest expectations were indicated for learning to search literature, to discuss scientifically and critically appraise literature (Additional file 2: Table S2). Expectations were lowest on acquiring understanding of research ethics. When students with the lowest and the highest ratings were compared, the greatest differences between the groups were in expectations on developing their oral and written communication skills and problem-solving ability (Additional file 2: Table S2). Regression analyses showed that there were no statistically significant differences between younger (< 22 years) and older (> 27 years) students, and students with and without previous research experience (data not shown). Those with a previous university degree (in subjects other than medicine) had lower expectations than students without a previous degree, especially for scientific writing, oral communication and interest for research (*p* < 0.001) (Additional file 2: Table S3). Moreover, female students had higher expectations than male students. Overall, students with basic science projects had the highest expectations, e.g., regarding participation in scientific discussions (*p* = 0.008) and becoming more interested in research (*p* = 0.003) (Additional file 2: Table S3). The only exception was learning statistics, on which students with epidemiological projects had the highest expectations (*p* < 0.001).

### Post-course rating of knowledge and skills

After the course, and in accordance with their expectations, students perceived they had learned skills needed to carry out a research project to a higher extent than they had acquired factual knowledge. The highest post-course ratings were for having developed the ability to search and critically appraise literature, as well as to write and discuss scientifically (Additional file 2: Table S2). However, the post-course ratings for these items were all lower than the expectations. Research ethics received the lowest total rating. Overall, the differences between students with the lowest and highest ratings diminished after the course and were greatest regarding research ethics and ability to participate in scientific discussions (Additional file 2: Table S2). However, for the group with lowest pre-course expectations the learning exceeded the expectations (data not shown). There was no statistically significant difference in students’ pre- and post-course interest in research, i.e. the group that was interested in research and rated that item high before the course did so also afterwards and the group with low interest continued to express this afterwards.

Regarding post-course ratings of skills related to future working life, the highest ratings were given to items pertaining to understanding the scientific basis of medicine, ability to follow current development of scientific knowledge and to critically integrate and use knowledge (Additional file 2: Table S4). Somewhat contradictory, the post-course ratings of items pertaining to own development regarding knowledge, skills and attitude were low (Additional file 2: Table S4). Moreover, the differences between low- and high-rating students were generally smaller for these transferable skills than they were for project-related knowledge and skills. The difference was smallest for the item understanding the scientific basis of medicine, which both groups rated high.

The regression analyses showed that there were no statistically significant differences between younger (<= 22 years) and older (> 27 years) students except for evaluation of own development in terms of knowledge (*p* = 0.006), skills (*p* = 0.008) and attitudes (*p* = 0.0017), which the younger students rated higher (data not shown). Compared to students without a previous university degree, those with a previous degree in subjects other than medicine rated their learning and development lower in areas such as participation in scientific discussion and scientific writing (*p* = 0.001) (Additional file 2: Table S5) but also in areas important in future working life such as following the development of knowledge. Compared to males, female students reported more positive learning experiences regarding ability to search literature (*p* = 0.021) and scientific writing (*p* = 0.044). Finally, the type of study also had an impact but there was no clear pattern. Those who did basic science projects reported higher learning in scientific discussions and scientific writing compared to those who did clinical projects, while those with projects categorized as *Other* rated their learning lower across most areas. Students who did epidemiological projects rated highest their learning to analyze complex phenomena (Additional file 2: Table S5). Previous research experience had no significant influence on any analyzed items (data not shown). The correlations between post-course ratings of learning and skills (Additional file 2: Table S6) showed no obvious patterns except that knowledge of statistics had a weak and problem-solving ability a relatively high correlation with all skills.

## Discussion

Research-related training is increasingly common in the basic curricula of medical schools, not least to promote awareness and use of a scientific approach in clinical work. In order to provide a learning environment that supports the necessary independent thinking and higher order skills, e.g. analysis and synthesis of data [15–17], it is essential to understand not only students’ learning experiences, but also their expectations on learning. This study is unusual in that it provides insights into students’ expectations and learning on a mandatory research project course, and it is thus not limited to students with explicit research interest. The expectations were highest regarding learning to search and critically appraise literature and to write scientifically. Overall, the post-course ratings showed that the expectations were not quite met. The gaps were greatest for oral communication, ability to participate in scientific discussions, and formulate hypotheses. Unexpectedly, research ethics received low ratings both before and after the course. Previous university degree had an impact on both the expectations and experienced learning.

The highest ratings, both for expectations as well as perceived gains, were for ability to search and critically appraise literature, which is in line with previous research [8], and encouraging since these skills are needed in evidence-based practice [18]. On the other hand, we found quite big negative gaps between expectations and learning for the items *participating in scientific discussions* and *formulating hypotheses*. The competencies needed to discuss science and formulate hypotheses include critical analysis and interpretation of data as well as ability to put results into a context of published literature [19]. Because the students had received very little pre-course training in these skills, we suggest that although the negative gap may reflect a lack of confidence, it might also reflect an insight that the skills indeed require more practice than the students had thought. It should also be noted that while scientific discussion was practiced on the seminars on the course, formulating hypotheses was not an explicit learning outcome, and as the students’ projects are provided by supervisors and reviewed by the coordinators, students themselves did in general not participate in formulating hypothesis of their own projects. We are not aware of any previous study addressing this particular skill. Interestingly, students with basic science projects gave slightly higher ratings regarding problem solving and scientific discussion. This may reflect the more dynamic development of such projects, in that hypotheses and questions may change from one week to another depending on experimental results. Similarly, the basic science research environment often offers ample opportunity to attend seminars and other formal and informal scientific discussions. Nevertheless, in view of the length of the course (20 weeks) and the live scientific projects, it is surprising that the post-course rating of these items remained low.

Biostatistics and epidemiology are essential tools in medical research and clinical decision-making [20]. Our students had high expectations on learning statistics, which is in line with Milic et al. [21] who showed that medical students have positive attitude to learning statistics. The gap between expectations and learning identified in the current study may be explained in part by the circumstance that the supervisors have the ultimate responsibility for organizing data collection, analysis and interpretation. Thus, the extent and methods of teaching statistics probably differed between projects. Another explanation for the gap could be the fact that some students selected descriptive, basic science or qualitative studies in which statistical analysis plays a lesser role. Moreover, statistics is a complex subject. Chiesi and Primi [22] reported a correlation between cognitive and non-cognitive factors that affect achievement in statistics; students with less competence in mathematics had less confidence, displayed more negative feelings, and considered statistics more difficult than did students with better mathematical competence. Thus, educators should aim at boosting students’ mathematical competence as well as attitudes to statistics to achieve best possible results [21]. On the other hand, Miles et al. [23] who investigated the views of practicing clinicians ended up with the recommendation that teaching statistics should not only be integrated in short research projects but also in the clinical subjects, making statistics practical and relevant already before the research projects are carried out. In addition, the teaching should focus more on interpretation and understanding the concepts rather than carrying out statistical calculations [23].

Similarly, students’ expectations on developing their skills in scientific writing were not entirely met. It is interesting that while the students had not received any focused training in scientific writing before the project course, they already before the course had a notion of the importance of a specific, scientific style of communication. This, however, did not extend to oral communication, since this item was rated relatively low both before and after the course. These results are in concordance with those of Burgoyne et al. [24]. Their survey comprised medical students from years 1 to 4 and showed that students perceived their scientific writing skills as higher than those in oral communication. The students in the present study got training in oral communication as the projects were presented in the format and style of standard scientific presentations during the three mandatory seminars. However, most students had had only one pre-course opportunity to present scientific topics orally, suggesting that they would expect even more training on the research project course. Other explanations for the the low ratings could be that students were too focused on the written report, which constituted the main component of the final assessment, or had limited knowledge or experience of their research area that affected their confidence and presentation skills. Finally, some students were perhaps already competent in presentation skills, through previous degree or research experience, and while they expected to improve these skills during their research project, they found that their previous level of ability was adequate. Haber and Lingard [25] showed in their ethnographic study that medical students regarded oral presentations as a rule-based, data-storage activity governed by “order” and “structure” while teachers viewed presentations as a flexible means of communication. The authors concluded that students learn oral presentation by trial and error rather than being taught an explicit mode, which may delay the development of effective communication skills. We agree with these authors [26] that teaching and learning oral presentation skills may be improved by systematic feedback, refining the instructions and emphasizing the relevance of these presentations for future clinical practice when students have to explain and adapt scientific information to patients and colleagues [2, 26].

A rather surprising finding was the low ratings for research ethics both before and after the course. Previous work has shown that medical students identified ethical values as an important characteristic of a good physician [27] but there are no studies assessing how research ethics have been grasped during scholarly projects. In view of the time limit of the projects, the students in the current study were not required to submit ethical applications for their projects, as these have to be submitted by supervisors before the projects can be started. Furthermore, some of the projects such as basic science studies without animal models do not need ethical approval. Nevertheless, by this stage in their education our students should be familiar with the ethical guidelines that regulate medical practice and research [28]. We recommend that, in order to demonstrate the role of ethics in modern research, the research project courses should encourage students’ active participation in ethical discussions and writing ethical applications as a learning activity, rather than using only theoretical teaching about laws or unfortunate cases from earlier research.

It has been shown that exposure to research may reinforce pre-existing interest in research and research career [29, 30]. In the current study, responses considering present and future interest in research were mixed. Interestingly, we found that having a previous university degree, i.e., some exposure to research, did not correlate with increased interest, or at least not in medical research. This finding is in line with a recent meta-analysis showing no significant correlation between a degree prior to medical school enrolment and subsequent research interest [3]. However, we also found that compared to students with a previous degree, the students without one became more interested in doing research in the future. Given that all university degrees in our country comprise a mandatory individual research project, it is plausible that students with a previous degree were already familiar with research and were more confident in their research skills, as described by Amgad et al. [3]. It is also possible that as students with a previous degree were older, they had had cause to carefully consider which career to focus on, and were set on one as a clinical practitioner [31]. Finally, the higher expectations among students without a degree could be due to having only a vague idea of what research is; however, we could not assess this possibility.

Based on our findings here and elsewhere [31, 32], we suggest several actions for helping medical students develop their scientific skills. Theory and practice regarding literature search should be introduced as early as possible, with stepwise increments in requirements on presenting short summaries or reviews of scientific reports, and later also notional project plans, all combined with feedback from senior reserachers and and peers. Journal club type seminars can be held within many different types of courses, and can be introduced fairly early in a context of instruction in biostatistics, epidemiology and study design. Research ethics may also be introduced and taught stepwise in parallel with, e.g., journal clubs, with increasing detail with regard to real-life dilemmas and situations.

It is a strength of this study that the participants were all in the same curricular stage (semester 7) and that the research project course was mandatory, wherefore the studied group was not limited to only those who already have a research interest. However, the fact that the students were all from the same institution limits the possibility to generalize the findings. Although the response rates were relatively high before and after the course, not all had filled in both questionnaires, wherefore the final response rate was moderate. Nevertheless, we feel the sample is of sufficient variety and size to provide relevant data. Finally, quantitative studies with self-reported measures are inexact and might be completed with qualitative ones to obtain more complete information or to diminish biases [33]. It should be noted that this study is not an evaluation of the research project course as such, but an evaluation probing the gap between what students considered important to learn and how they rated their learning and we can assume that the students are capable of such rating.

## Conclusions

Students were found to already before the course appreciate the role of scientific core skills such as searching and appraising literature, and scientific writing, whereas statistics, hypothesis formulation, oral communication and in particular research ethics received lower ratings. The distribution of post-course ratings of achieved learning were similar, but lower than the expectation ratings. We suggest that students should be actively involved in ethical discussions in order to appreciate the role of ethics in modern research. Moreover, oral communication should be trained repeatedly as an important role of the doctors is to communicate scientific information to patients and non-experts. Factors that affect interest in medical research need to be investigated in more detail.

## Additional files

Additional file 1: Questionnaires. Questionnaires before and after the research projects. (DOC 36 kb)

Additional file 2: Tables S2 to S6. Tables with data (DOCX 40 kb)

## Abbreviations

EBM: Evidence-based medicine
MD: Medical Doctor
MeSH: Medical Subject Headings
PhD: Doctor of Philosophy
