# Supplementary material for: Does reality meet expectations? An analysis of medical students’ expectations and perceived learning during mandatory research projects
Source: BMC Med Educ. 2019 Mar 29;19:93. doi: 10.1186/s12909-019-1526-x (PMC6439984; doi:10.1186/s12909-019-1526-x)
Supplement: Supplementary file 1 — Questionnaires. Questionnaires before and after the research projects. (DOC 36 kb) [file 12909_2019_1526_MOESM1_ESM.doc]

**Medical students’ expectations before the mandatory research project**

1. Name
2. Age
3. Gender
4. Do you have a previous university degree? Yes/No

If yes, which degree?

1. Have you previously studied at a university without taking a degree? Yes/No

If yes, for how long? (the number of semesters)

If yes, in which area?

1. Have you previously been admitted to doctoral education? Yes/No
2. If yes, for how long you have been carrying out research (in months)?
3. Have you any other previous experience in carrying out research (not doctoral studies nor scholarly projects*) before the research project course?

**Other research experience can be e.g. work in a laboratory during summer holidays or studies that have led or will lead to a research report that you write alone or together with others.*Yes/No

1. If yes, how long is your experience (in months)?
2. In the following we ask you to rate (on a 5-point Likert scale) what are your personal expectations for learning during the course (1=none at all; 2=To a small extent; 3=To some extent; 4=To a great extent; 5= To a very great extent).
   1. I will develop my ability to search literature
   2. I will develop my ability to critically appraise literature
   3. I will develop my ability to participate in scientific discussions
   4. I will develop my ability in scientific writing
   5. I will learn more statistics
   6. I will develop my ability to formulate scientific hypothesis
   7. I will develop my problem-solving ability
   8. I will develop my ability in oral communication
   9. I will become more interested in research
   10. I will learn research ethics
3. Did the questionnaire evoke any thoughts or reactions?

**Medical students’ perceptions of learning during the mandatory research project**

1. Name
2. Age
3. Gender
4. In the following we ask you to rate (on a 5-point Likert scale) your perceptions of learning during the course (1=none at all; 2=To a small extent; 3=To some extent;4=To a great extent; 5= To a very great extent).
   1. I have developed my ability to search literature
   2. I have developed my ability to critically appraise literature
   3. I have developed my ability to participate in scientific discussions
   4. I have developed my ability in scientific writing
   5. I will learn more statistics
   6. I have developed my ability to formulate scientific hypothesis
   7. I have developed my problem-solving ability
   8. I have developed my ability in oral communication
   9. I have become more interested in research
   10. I have learned research ethics
5. In the following we ask you to rate (on a 5-point Likert scale) your perceptions of learning during the course (1=none at all; 2=To a small extent; 3=To some extent;4=To a great extent; 5= To a very great extent). I have learnt to…
   1. Understand the scientific basis of medicine
   2. Follow the development of scientific knowledge
   3. Independently and critically integrate knowledge
   4. Identify the need for additional knowledge and develop my competencies
   5. Critically and systematically analyze complex phenomena
   6. Work with high requirements for independence and development of care
   7. Face changes in working life
   8. Evaluate my own development in terms of knowledge
   9. Evaluate my own development in terms of attitude
   10. Evaluate my own development in terms of skills
6. Did the questionnaire evoke any thoughts or reactions?
