# Supplementary material for: Does reality meet expectations? An analysis of medical students’ expectations and perceived learning during mandatory research projects
Source: BMC Med Educ. 2019 Mar 29;19:93. doi: 10.1186/s12909-019-1526-x (PMC6439984; doi:10.1186/s12909-019-1526-x)
Supplement: Supplementary file 2 — Tables S2 to S6. Tables with data (DOCX 40 kb) [file 12909_2019_1526_MOESM2_ESM.docx]

**Table S2** Ratings (mean, SD) for expectations and perceptions of learning before and after a research project course *(n=358).*

|  | **Ratings for expectations before the course**  **Mean (SD)** | | | **Ratings of learning after the course**  **Mean (SD)** | | | **∆ Before vs after** | |
| --- | --- | --- | --- | --- | --- | --- | --- | --- |
| **Items** | **All students** | **Students with lowest ratings** | **Students with highest ratings** | **All students** | **Students with lowest ratings** | **Students with highest ratings** | **All students** | **P-value** |
| Search literature | 4.2 (0.9) | 3.2 (1.4) | 4.5 (0.7) | 4.0 (0.9) | 3.3 (1.1) | 4.3 (0.7) | -0.2 | ns |
| Critically appraise literature | 4.1 (0.9) | 3.1 (1.4) | 4.4 (0.8) | 3.8 (1.0) | 3.2 (1.0) | 4.0 (1.0) | -0.3 | <0.0001 |
| Participate in scientific discussions | 4.1 (0.9) | 2.9 (1.0) | 4.4 (0.8) | 3.6 (1.0) | 2.8 (1.1) | 3.9 (0.9) | -0.5 | <0.0001 |
| Scientific writing | 4.1 (1.0) | 2.8 (1.5) | 4.4 (0.9) | 3.9 (1.0) | 3.1 (1.2) | 4.1 (0.9) | -0.2 | <0.0001 |
| Statistics | 4.0 (1.0) | 3.5 (1.3) | 4.4 (0.8) | 3.7 (1.1) | 3.1 (1.4) | 3.9 (1.1) | -0.3 | <0.0001 |
| Formulate a hypothesis | 3.8 (0.9) | 2.6 (1.1) | 4.1 (0.9) | 3.4 (1.1) | 2.6 (1.1) | 3.6 (1.0) | -0.4 | <0.0001 |
| Solve problems | 3.7 (1.0) | 2.3 (1.2) | 4.0 (0.9) | 3.4 (1.0) | 2.4 (1.1) | 3.6 (1.0) | -0.3 | <0.0001 |
| Oral communication | 3.6 (1.0) | 2.3 (1.3) | 3.9 (1.0) | 3.1 (1.1) | 2.4 (1.3) | 3.3 (1.1) | -0.5 | <0.0001 |
| Become more interested in research | 3.4 (1.1) | 2.4 (1.4) | 3.8 (1.0) | 3.2 (1.4) | 2.5 (1.5) | 3.5 (1.3) | -0.2 | ns |
| Research ethics | 3.1 (1.0) | 1.8 (1.0) | 3.3 (1.0) | 3.0 (1.1) | 2.1 (1.4) | 3.2 (1.1) | -0.1 | ns |

Note. SD Standard Deviation. Expectations and perceptions were graded on a Likert scale ranging from 1 (low priority, not at all); 2 (to small extent); 3 (to some extent); 4 (to large extent); and 5 (to very large extent). Students with lowest ratings were defined as having least two ratings of 1, and students with highest ratings had at least two ratings of 5. **∆** mean difference between expectations and learning. P-value shows the significance level of change.

**Table S3** Factors associated with students’ expectations during research project course *(n= 358)*.

|  | **Gender** | | | **Previous university degree** | | | **Type of study** | | | | | | |
| --- | --- | --- | --- | --- | --- | --- | --- | --- | --- | --- | --- | --- | --- |
|  |  | | |  | | | **Basic science** | | **Epidemiological** | | **Other** | |  |
| **Expectations before the course** | **OR** | **95% CI** | **p-value** | **OR** | **95% CI** | **p-value** | **OR** | **95% CI** | **OR** | **95% CI** | **OR** | **95% CI** | **p-value** |
| Search literature | 1.6 | 1.1 – 2.5 | 0.024 | 0.5 | 0.2 – 0.9 | 0.034 | 0.9 | 0.5 – 1.6 | 1.2 | 0.6 – 2.5 | 1.1 | 0.5 – 2.5 | 0.92 |
| Critically appraise literature | 1.5 | 1.0 – 2.2 | 0.081 | 0.4 | 0.2 – 0.8 | 0.010 | 1.2 | 0.7 – 1.9 | 1.1 | 0.5 – 2.2 | 0.8 | 0.4 – 1.8 | 0.86 |
| Participate in scientific discussions | 1.4 | 0.9 – 2.1 | 0.15 | 0.4 | 0.2 – 0.8 | 0.009 | 1.5 | 0.9 – 2.6 | 0.7 | 0.3 – 1.3 | 0.3 | 0.1 – 0.8 | 0.008 |
| Scientific writing | 1.9 | 1.2 – 2.9 | 0.003 | 0.2 | 0.1 – 0.4 | <0.001 | 1.4 | 0.8 – 2.4 | 1.0 | 0.5 – 1.9 | 0.3 | 0.1 – 0.7 | 0.015 |
| Statistics | 1.6 | 1.0 – 2.4 | 0.041 | 0.4 | 0.2 – 0.9 | 0.025 | 0.6 | 0.4 – 1.1 | 2.0 | 1.0 – 4.2 | 0.3 | 0.1 – 0.6 | <0.001 |
| Formulate a hypothesis | 1.2 | 0.8 – 1.7 | 0.50 | 0.4 | 0.2 – 0.7 | 0.005 | 1.4 | 0.8 – 2.3 | 1.9 | 1.0 – 3.7 | 0.7 | 0.3 – 1.7 | 0.15 |
| Solve problems | 1.8 | 1.2 – 2.7 | 0.008 | 0.4 | 0.2 – 0.7 | 0.004 | 2.1 | 1.2 – 3.5 | 1.7 | 0.8 – 3.6 | 0.9 | 0.4 – 2.0 | 0.026 |
| Oral communication | 2.3 | 1.5 – 3.6 | <0.001 | 0.2 | 0.1 – 0.4 | <0.001 | 1.6 | 1.0 – 2.7 | 1.3 | 0.6 – 2.5 | 0.8 | 0.4 – 1.6 | 0.20 |
| Become more interested in research | 1.7 | 1.1 – 2.6 | 0.013 | 0.3 | 0.1 – 0.6 | <0.001 | 2.3 | 1.4 – 3.8 | 1.5 | 0.8 – 3.0 | 0.6 | 0.2 – 1.3 | 0.003 |
| Research ethics | 1.6 | 1.1 – 2.5 | 0.025 | 0.6 | 0.3 – 1.2 | 0.150 | 1.1 | 0.6 – 1.8 | 1.2 | 0.6 – 2.3 | 0.4 | 0.2 – 0.9 | 0.16 |

**Table S4** Students´ ratings (mean, SD) of perceived gains in generic skills related to future working life (*n =358*)*.*

|  | **All students**  **Mean (SD)** | **Students with lowest ratings**  **Mean (SD)** | **Students with highest ratings**  **Mean (SD)** |
| --- | --- | --- | --- |
| Understand the scientific basis of medicine | 3.8 (0.9) | 3.5 (1.2) | 4.0 (0.8) |
| Follow the development of scientific knowledge | 3.5 (0.9) | 3.0 (1.2) | 3.7 (0.8) |
| Independently and critically integrate knowledge | 3.5 (1.0) | 2.7 (1.2) | 3.8 (0.9) |
| Identify the need for additional knowledge and develop my competencies | 3.4 (1.0) | 2.9 (1.2) | 3.6 (0.9) |
| Critically and systematically analyze complex phenomena | 3.4 (1.0) | 2.7 (1.1) | 3.6 (0.9) |
| Work with high requirements for independence and development of care | 3.1 (1.1) | 2.5 (1.5) | 3.4 (1.0) |
| Face changes in working life | 3.1 (1.1) | 2.7 (1.5) | 3.3 (1.1) |
| Evaluate my own development in terms of knowledge | 2.9 (1.1) | 2.2 (1.3) | 3.1 (1.1) |
| Evaluate my own development in terms of attitude | 2.8 (1.1) | 2.2 (1.2) | 3.0 (1.1) |
| Evaluate my own development in terms of skills | 2.7 (1.2) | 2.2 (1.3) | 3.0 (1.2) |

Note: SD Standard Deviation. The statements were graded on Likert scale of 1 (not at all); 2 (to small extent); 3 (to some extent); 4 (to large extent);

and 5 (to very large extent). Students with lowest ratings were defined as having least two ratings of 1, and students with highest ratings had at least

two ratings of 5.

**Table S5** Factors associated with students’ learning during research project course *(n= 358)*.

|  | **Gender** | | | **Previous university degree** | | | **Type of study** | | | | | | |
| --- | --- | --- | --- | --- | --- | --- | --- | --- | --- | --- | --- | --- | --- |
|  |  | | |  | | | **Basic science** | | **Epidemiological** | | **Other** | |  |
|  | **OR** | **95% CI** | **p-value** | **OR** | **95% CI** | **p-value** | **OR** | **95% CI** | **OR** | **95% CI** | **OR** | **95% CI** | **p-value** |
| **Learning after the course** |  |  |  |  |  |  |  |  |  |  |  |  |  |
| Search literature | 1.6 | 1.1 – 2.5 | 0.021 | 0.4 | 0.2 – 0.8 | 0.006 | 0.9 | 0.5 – 1.6 | 1.2 | 0.6 – 2.5 | 1.1 | 0.5 – 2.5 | 0.92 |
| Critically appraise literature | 1.4 | 0.9 – 2.1 | 0.13 | 0.4 | 0.2 – 0.7 | 0.006 | 1.2 | 0.7 – 1.9 | 1.1 | 0.5 – 2.2 | 0.8 | 0.4 – 1.8 | 0.86 |
| Participate in scientific discussions | 1.1 | 0.8 – 1.7 | 0.52 | 0.3 | 0.2 – 0.6 | 0.001 | 1.5 | 0.9 – 2.6 | 0.7 | 0.3 – 1.3 | 0.3 | 0.1 – 0.8 | 0.008 |
| Scientific writing | 1.5 | 1.0 – 2.3 | 0.044 | 0.3 | 0.1 – 0.6 | 0.001 | 1.4 | 0.8 – 2.4 | 1.0 | 0.5 – 1.9 | 0.3 | 0.1 – 0.7 | 0.015 |
| Statistics | 1.1 | 0.8 – 1.7 | 0.55 | 0.5 | 0.2 – 1.0 | 0.048 | 0.6 | 0.4 – 1.1 | 2.0 | 1.0 – 4.2 | 0.3 | 0.1 – 0.6 | <0.001 |
| Formulate a hypothesis | 1.2 | 0.8 – 1.8 | 0.48 | 0.5 | 0.2 – 0.9 | 0.034 | 1.4 | 0.8 – 2.3 | 1.9 | 1.0 – 3.7 | 0.7 | 0.3 – 1.7 | 0.15 |
| Solve problems | 1.5 | 1.0 – 2.3 | 0.056 | 0.4 | 0.2 – 0.7 | 0.004 | 2.1 | 1.2 – 3.5 | 1.7 | 0.8 – 3.6 | 0.9 | 0.4 – 2.0 | 0.026 |
| Oral communication | 1.5 | 1.0 – 2.2 | 0.077 | 0.4 | 0.2 – 0.9 | 0.021 | 1.6 | 1.0 – 2.7 | 1.3 | 0.6 – 2.5 | 0.8 | 0.4 – 1.6 | 0.20 |
| Become more interested in research | 1.4 | 0.9 – 2.1 | 0.12 | 0.5 | 0.3 – 1.0 | 0.049 | 2.3 | 1.4 – 3.8 | 1.5 | 0.8 – 3.0 | 0.6 | 0.2 – 1.3 | 0.003 |
| Research ethics | 1.5 | 1.0 – 2.2 | 0.070 | 0.4 | 0.2 – 0.8 | 0.010 | 1.1 | 0.6 – 1.8 | 1.2 | 0.6 – 2.3 | 0.4 | 0.2 – 0.9 | 0.16 |
| **Gains in skills related to future working life** |  |  |  |  |  |  |  |  |  |  |  |  |  |
| Understand the scientific basis of medicine | 1.7 | 1.0 – 2.9 | 0.052 | 0.4 | 0.2 – 0.9 | 0.036 | 1.1 | 0.7 – 1.9 | 0.9 | 0.4 – 1.8 | 0.5 | 0.2 – 1.2 | 0.35 |
| Follow the development of scientific knowledge | 1.3 | 0.8 – 2.2 | 0.35 | 0.3 | 0.1 – 0.7 | 0.008 | 0.8 | 0.5 – 1.3 | 1.3 | 0.7 – 2.6 | 0.4 | 0.2 – 1.0 | 0.16 |
| Independently and critically integrate knowledge | 1.5 | 0.9 – 2.5 | 0.15 | 0.5 | 0.2 – 1.2 | 0.11 | 1.5 | 0.9 – 2.5 | 1.0 | 0.5 – 2.0 | 0.4 | 0.2 – 0.9 | 0.045 |
| Identify the need for additional knowledge and develop my competencies | 1.4 | 0.8 – 2.4 | 0.23 | 0.8 | 0.3 – 1.8 | 0.56 | 1.6 | 1.0 – 2.7 | 1.1 | 0.5 – 2.1 | 0.3 | 0.1 – 0.6 | 0.002 |
| Critically and systematically analyze complex phenomena | 1.1 | 0.6 – 1.8 | 0.82 | 0.5 | 0.2 – 1.2 | 0.14 | 0.5 | 0.3 – 0.7 | 1.5 | 0.8 – 3.1 | 0.4 | 0.2 – 1.1 | 0.001 |
| Work with high requirements for independence/development of care | 1.2 | 0.7 – 2.1 | 0.42 | 0.6 | 0.2 – 1.3 | 0.16 | 1.5 | 0.9 – 2.6 | 1.2 | 0.6 – 2.3 | 0.5 | 0.2 – 1.2 | 0.11 |
| Face changes in working life | 1.5 | 0.9 – 2.5 | 0.15 | 0.4 | 0.2 – 0.8 | 0.018 | 2.0 | 1.2 – 3.4 | 1.7 | 0.8 – 3.4 | 0.8 | 0.4 – 1.9 | 0.029 |
| Evaluate my own development in terms of knowledge | 1.0 | 0.7 – 1.5 | 0.97 | 0.3 | 0.2 – 0.7 | 0.002 | 1.4 | 0.8 – 2.3 | 1.1 | 0.6 – 2.3 | 0.5 | 0.2 – 1.2 | 0.20 |
| Evaluate my own development in terms of attitude | 1.0 | 0.6 – 1.5 | 0.90 | 0.5 | 0.2 – 0.9 | 0.021 | 1.4 | 0.9 – 2.3 | 1.4 | 0.7 – 2.7 | 0.4 | 0.2 – 0.9 | 0.036 |
| Evaluate my own development in terms of skills | 1.0 | 0.7 – 1.5 | 0.93 | 0.4 | 0.2 – 0.8 | 0.009 | 1.1 | 0.7 – 1.8 | 1.0 | 0.5 – 2.1 | 0.4 | 0.2 – 0.8 | 0.071 |

**Table S6** Correlation coefficient matrix between post-course ratings of learning and skills *(n= 358)*.

|  | Understand the scientific basis of medicine | Follow the development of scientific knowledge | Independently and critically integrate knowledge | Identify needs for additional knowledge and to develop competencies | Critically and systematically analyze complex phenomena | Work with high demands on independence and development of care | Face changes in working life | Evaluate own development in terms of | | |
| --- | --- | --- | --- | --- | --- | --- | --- | --- | --- | --- |
|  |  |  |  |  |  |  |  | Knowledge | Skills | Attitude |
| Search literature | 0.41 | 0.43 | 0.38 | 0.40 | 0.38 | 0.34 | 0.24 | 0.33 | 0.29 | 0.30 |
| Critically appraise literature | 0.39 | 0.43 | 0.42 | 0.30 | 0.43 | 0.34 | 0.23 | 0.37 | 0.36 | 0.33 |
| Participate in scientific discussions | 0.46 | 0.45 | 0.54 | 0.51 | 0.51 | 0.46 | 0.40 | 0.52 | 0.48 | 0.49 |
| Scientific writing | 0.37 | 0.41 | 0.45 | 0.40 | 0.46 | 0.44 | 0.34 | 0.42 | 0.41 | 0.39 |
| Statistics | 0.26 | 0.24 | 0.30 | 0.20 | 0.28 | 0.23 | 0.14 | 0.24 | 0.21 | 0.24 |
| Formulate a hypothesis | 0.40 | 0.39 | 0.44 | 0.43 | 0.48 | 0.36 | 0.34 | 0.46 | 0.48 | 0.48 |
| Solve problems | 0.40 | 0.43 | 0.55 | 0.50 | 0.62 | 0.52 | 0.50 | 0.56 | 0.56 | 0.52 |
| Oral communications | 0.27 | 0.34 | 0.45 | 0.39 | 0.41 | 0.40 | 0.45 | 0.45 | 0.44 | 0.44 |
| Become more interested in research | 0.37 | 0.38 | 0.38 | 0.41 | 0.35 | 0.41 | 0.33 | 0.39 | 0.40 | 0.41 |
| Research ethics | 0.32 | 0.29 | 0.43 | 0.42 | 0.41 | 0.32 | 0.42 | 0.45 | 0.45 | 0.48 |
